# Supplementary material for: Exosomes Derived from Induced and Wharton’s Jelly-Derived Mesenchymal Stem Cells Promote Senescence-like Features and Migration in Cancer Cells
Source: Int J Mol Sci. 2025 Jun 26;26(13):6178. doi: 10.3390/ijms26136178 (PMC12249808; doi:10.3390/ijms26136178)
Supplement: Supplementary file 1 [file ijms-26-06178-s001.zip › ijms-3665860-supplementary.pdf]

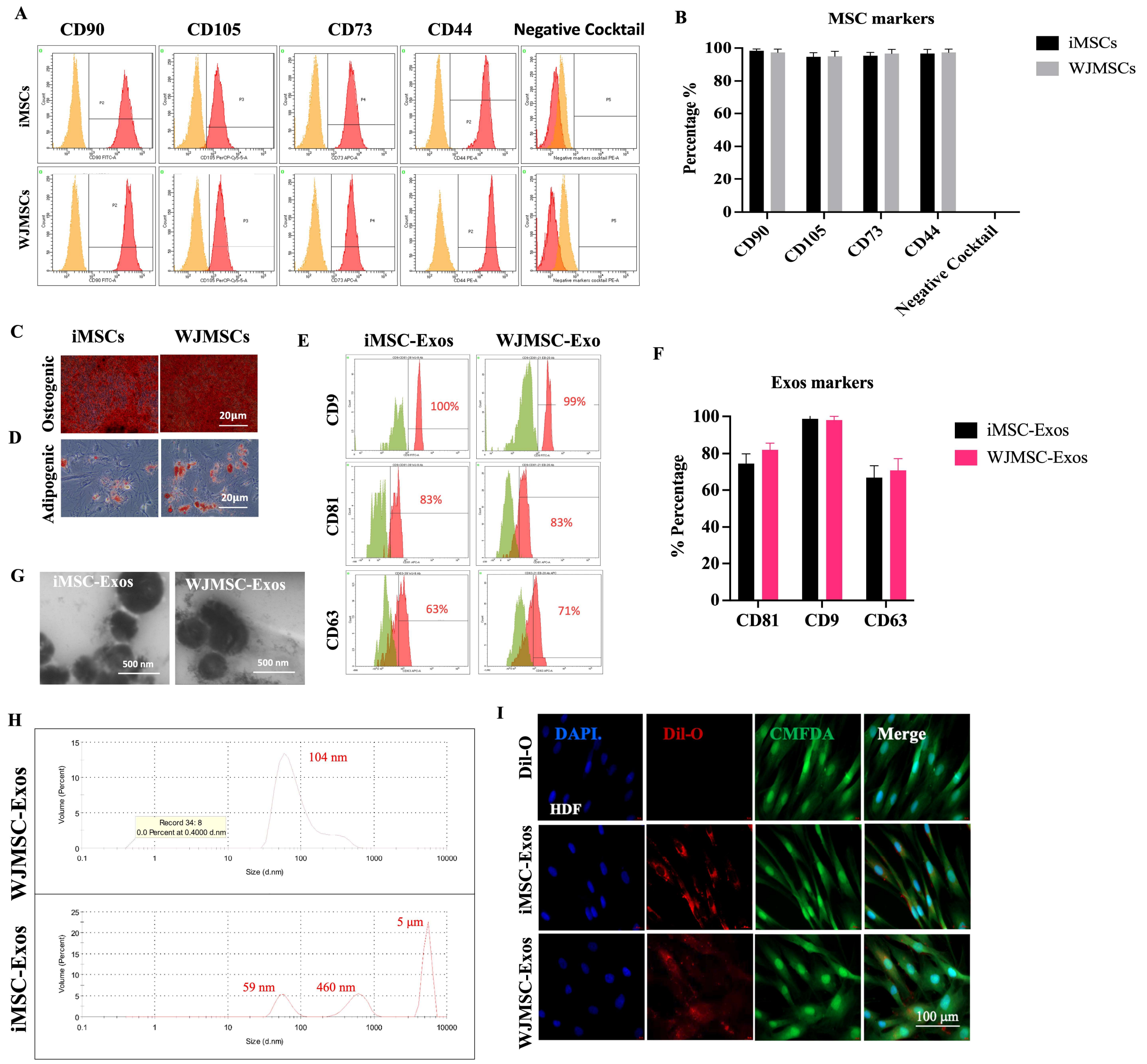

Figure S1. Characterization of iMSCs and WJMSCs and their relative exosomes. (A) Histograms for flow cytometric analysis of MSC surface markers. (B) The graph shows the expression levels of hMSC surface markers for iMSCs and WJMSCs. (C) Alizarin Red staining indicates the presence of calcium deposits in the osteogenically differentiated iMSCs and WJMSCs, while Oil Red O staining highlights fat vacuoles in the adipogenically differentiated cells. The iMSCs are shown in the lower left (D), and the WJMSCs are in the lower right. (E) Representative images of flow cytometric histograms displaying the presence of exosome surface markers (CD9, CD81, and CD63). (F) Statistical analysis comparing the percentages of surface markers in iMSC-Exos versus WJMSC-Exos. (G) Representative TEM images of WJMSC-Exos (left) and iMSC-Exos (right), with a scale bar of 500 nm and 1 µm respectively. (H) Size distribution measurements obtained via DLS for isolated WJMSC-Exos (upper) and iMSC-Exos (lower). (I) Internalization of iMSC- and WJMSC-derived exosomes by human dermal fibroblasts (HDF). Cells were stained with CMFDA (green) for the cytoplasm and DAPI (blue) for nuclei. Successful uptake of iMSC- and WJMSC-derived exosomes labeled with DiI (red) was confirmed in both cell types.

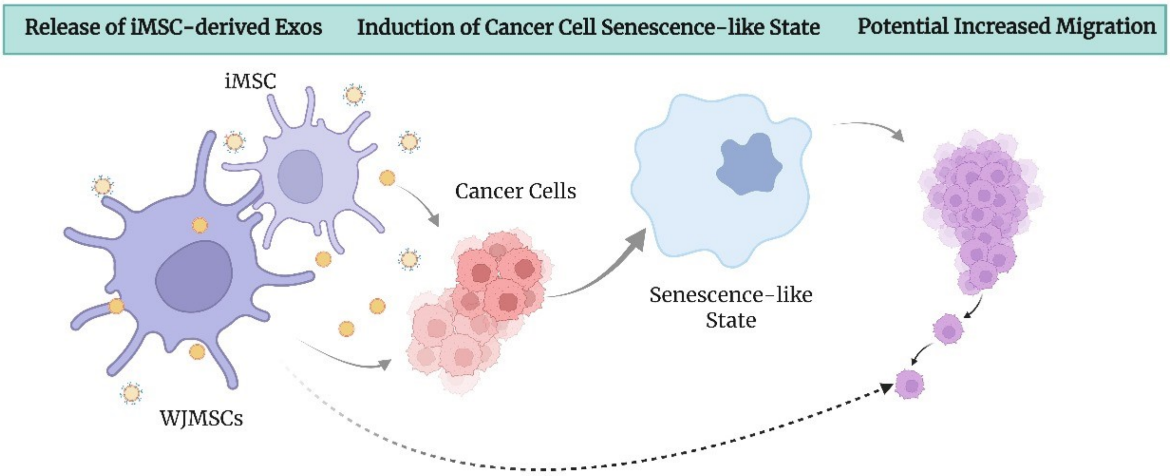

Figure S2. Schematic summary of exosome-induced senescence and migration effects in cancer cells. iMSC- and WJMSC-derived exosomes induced senescence in MCF7 and A549 cancer cells. However, only WJMSC-Exos promoted migration through a senescence-like state in these cancer cells, while iMSC-Exos did not affect migration, highlighting a key functional difference between the two exosome types in modulating cancer cell behavior. Created in https://BioRender.com

Table S1. Primers list for the quantification of senescence-associated genes using RT-qPCR

Gene	Sequence (5′-3′)	Annealing Temperature
P53^{Cip1}	F: 5′GTTCCGAGAGCTGAATGAGG-3′<br>R: 5′TCTGAGTCAGGCCCTTCTGT-3′	63.1°C
P21^{Cip1}	F: 5'GAGGCCGGGATGAGTTGGGAGGAG-3'<br>R: 5'CAGCCGGCGTTTGGAGTGGTAGAA-3'	63.1°C
CXCL8	F: 5′AAGAGCCAGGAAGAAACCACC-3′<br>R: 5′CTGCAGAAATCAGGAAGGCTG-3′	60°C
IL6	F: 5′-AGACTTCACAGAGGATACCACCCAC-3′<br>R: 5′-CAATCAGAATTGCCATTGCACAA-3′	63.1°C
TNF-α	F: 5′-TCTTCTCGAACCCCGAGTGA<br>F: 5′-CCTCTGATGGCACCACCAG	60°C
TGF-β1	F: 5′-CAGCAACAATTCCTGGCGATA<br>F: 5′-AAGGCGAAAGCCCTCAATTT	60°C
GAPDH	F: 5′CCTGTTCGACAGTCAGCCG-3′<br>R: 5′CGACCAAATCCGTTGACTCC-3′	60°C
